# Identification of hub gene for the pathogenic mechanism and diagnosis of MASLD by enhanced bioinformatics analysis and machine learning

**DOI:** 10.1371/journal.pone.0324972

**Published:** 2025-05-28

**Authors:** Hong Lu, Ziyong Mao, Mengyao Zheng, Min Zhang, Heqing Huang, Yiling Chen, Long Lv, Zutao Chen

**Affiliations:** 1 Infectious Disease Department, the First Affiliated Hospital of Soochow University, Suzhou, Jiangsu Province, China; 2 BamRock Research Department, Suzhou BamRock Biotechnology Ltd., Suzhou, Jiangsu Province, China; 3 College of Life Sciences, Qufu Normal University, Qufu, Shandong Province, China; 4 Department of Biological Sciences, University at Albany, Albany, New York, United States of America; 5 Central Laboratory, The Affiliated Gaochun Hospital of Jiangsu University, Nanjing, Jiangsu Province, China; 6 MOE Key Laboratory of Geriatric Diseases and Immunology, Suzhou Key Laboratory of Pathogen Bioscience and Anti-infective Medicine, the First Affiliated Hospital of Soochow University, Suzhou, Jiangsu Province, China; 7 Infectious Disease Department, the Fourth Affiliated Hospital of Soochow University, Suzhou, Jiangsu Province, China; University of Connecticut, UNITED STATES OF AMERICA

## Abstract

Metabolic dysfunction-associated steatotic liver disease (MASLD) is a heterogeneous disease caused by multiple etiologies. It is characterized by excessive fat accumulation in the liver. Without intervention, MASLD can progress from steatosis to metabolic dysfunction-associated steatohepatitis (MASH), fibrosis and even to cirrhosis and hepatocellular carcinoma. However, the pathogenesis of MASH and the mechanism underlying the development of fibrosis remain poorly understood, posing challenges for accurate diagnosis of MASH and fibrosis. In this study, we analyzed tissue RNA-seq data and clinical information of healthy individuals and MASLD patients from multiple datasets, the key genes and pathways involved in the occurrence and progression of MASLD, MASH, and fibrosis were screened respectively. Our findings reveal that the development of MASLD, MASH and fibrosis is associated with lipid metabolism processes. Based on the RNA expression profiles of identified hub genes, we established three alternative diagnostic models for MASLD, MASH, and fibrosis. These models demonstrated excellent performance in the diagnosis of MASLD, MASH, and fibrosis, with AUC values exceeding 0.9, implicating its potential clinical values in disease diagnosis.

## Introduction

MASLD is a complex process and closely linked to cardiometabolic disease, whilst excluding alcohol, genetic factors and other causes as contributors to liver disease [1–3]. As a result of the diabetes and metabolic syndrome epidemics, MASLD has risen to the top of the list of chronic liver diseases worldwide. The spectrum of MASLD ranges from initial metabolic dysfunction-associated fatty liver to MASH, and advanced conditions such as MASH-associated cirrhosis and hepatocellular carcinoma [4]. However, the disease progression is poorly understood.

This research builds on previous research data on metabolic liver disease, where it is was previously known as non-alcoholic fatty liver disease (NAFLD) [5]. It is advocated that the severity evaluation of MASLD is based on inflammation activity rather than the presence or absence of inflammation. The NAFLD Activity Score (NAS) system remains in use, evaluating histologic features into three distinct categories: lobular inflammation (0–3), steatosis (0–3), and ballooning (0–2) [6]. MASH is diagnosed with a NAS value of 5 or greater, a NAS value lower than 3 cannot be diagnosed with MASH. Currently, studies have found that an increase in NAS score is associated with the progression of liver fibrosis in the presence of steatohepatitis, while a decrease in NAS score is associated with resolution of fibrosis. Chronic inflammation activates the immune system and releases a large amount of inflammatory mediators (such as cytokines and chemokines). These substances stimulate the activation of hepatic stellate cells (HSCs), which in turn secrete excessive extracellular matrix (ECM), forming fibrous scars [7–9]. In mouse models of fatty liver disease, the dysregulation of some key genes has been shown to modulate hepatic inflammation and lipid accumulation, thereby contributing to the development of MASLD and fibrosis [31,32]. However, there is inherent difference between murine and human pathophysiology. The hub genes and pathways that involved in the development and progression of human MASLD, MASH and fibrosis remain unclear.

The histology of liver biopsy remains the gold standard for diagnosis. However, biopsy driven diagnosis has its limitations, namely bias and variable interpretation. Histological analysis of biopsy specimens requires experience and skill, but there is still a subjective bias that tends to vary within and between observers. For example, the agreement of liver pathologists on the diagnosis of fibrosis staging within observers is 60% ~ 90%, and the consensus among observers is 70% ~ 90% [10].

In this study, we employed an integrative analysis of multiple RNA-seq datasets to identify key genes and pathways implicated in the onset and progression of MASLD, MASH, and fibrosis. Through bioinformatic modeling and functional annotation, we investigated the biological roles and mechanistic contributions of these genes. Next, we explore whether there is a core set of genes and pathways that play a key role in the above process. Finally, alternative diagnostic models for MASLD, MASH and fibrosis were established through RNA expression profiles of liver tissue samples using a small set of hub genes, respectively. The implementation of these models may facilitate the standardization, objectification, and automation of diagnostic process. The results of this study are expected to provide valuable references for further research on MASLD disease mechanisms, diagnosis, and discovery of drug therapeutic targets.

## Methods

### Data collection and clinical information analysis

Gene expression data and clinical information of 346 MASLD and 226 normal liver tissues were acquired for model training from the laboratory data porta of professor Huang (http://www.gepliver.org/#/download) [11], including GepLiver-bulk-06 (GSE130970_MASLD), GepLiver-bulk-07 (GSE135251_ MASLD) and GepLiver-bulk-08 (GSE162694_MASLD), and GepLiver-bulk-01 (GTEx database_Normal). Several independent MASLD datasets (GepLiver-bulk-05_GSE126848, GepLiver-bulk-09_GSE167523, GepLiver-bulk-13_E-MTAB-6863) are used to verify our model. The detailed source and population demographics (sex and age) are described (see S1 File, Supplemental Digital Content, showed source and clinical information). The Pearson correlation coefficient was calculated to check the correlation between population demographics factors (sex and age) and MASH or fibrosis by using SPSS software (S6 File). The specific processing flow is described in detail in “GepLiver: a dynamic, integrative liver expression atlas spanning developmental stages and liver disease phases, figshare”. Our study used publicly available data, therefore, ethical IRB approval is not required.

### Differential expression analyses

In order to explore the differential expression genes (DEGs) that related to MASLD, MASH and fibrosis respectively, we divided the data into two distinct groups according to the following three distinct methods:

(1) To explore the DEGs associated with MASLD, the MASLD patients composed the disease group and the healthy individuals composed the control group.
(2) In order to investigate the DEGs associated with MASH, MASLD patients with NAS ≥ 5 were placed in disease group and MASLD patients with NAS < 3 composed the control group.
(3) To examine the DEGs associated with fibrosis, MASLD patients with a fibrosis stage > 0 were placed in disease group and MASLD without fibrosis (stage = 0) composed the control group. To reduce variability in fibrosis assessment, we investigated the inflammation difference between the fibrosis and control groups, and we confirmed that there is no inflammation-related bias.

Because our aim is to investigate the genes involved in fibrosis within the context of liver inflammation. Therefore, we excluded patients who lacked liver inflammation. Given that the majority of patients labeled as MAFLD in the GSE135251 database did not exhibit liver inflammation, we excluded these MAFLD patients from our analysis. Similarly, patients with an inflammation grade of 0 in the GSE130970 database were also excluded. It is noteworthy that the GSE162694 database was not considered for the fibrosis group analysis due to the absence of specific inflammatory information for individual patients.

The RNA-Seq data of liver tissues were analyzed using the Limma package of R language (http://www.bioconductor.org/packages/release/bioc/html/limma.html) to identify DEGs. The data from Gepliver database are preprocessed and there is no missing data. There were no outliers. The low-expression genes (expression level <1) were filtered out. The voom method was used to analyze DEGs: (1) the raw counts were converted to log2 CPM (counts per million reads), all counts were added by 0.5 to avoid taking logarithmic zeros; (2) the logCPM value matrix was normalized, the Non-Linear Least-Square Minimization and Compute Contrasts from Linear Model Fit was used to fit and compare the data; (3) the compared model was transformed into an empirical Bayesian model by eBayes, and the variance part of the t-test was adjusted. Finally, logFC, average expression, p value, t, q. value and B value were extracted. DEGs were selected based on the following criteria: q-value < 0.05 and |log_2_ Fold Change| (|log_2_ FC|) > 1. Heatmaps were generated using pheatmap package and volcano plots were also conducted in *R* software.

### Correlation analysis between gene expression and clinical phenotype

To examine the correlation between continuous variables (gene expression) and discrete variables (e.g., NAS or fibrosis stage), we used the ordinal logistic regression. The “polr” function in the R package MASS was used to conduct this analysis. The pseudo R square was calculated using the likelihood ratio method:


$$\mathrm{\backslash [}R^{2}=\frac{1-\left(logLik(model)\right)}{\left(logLik(nullmodel)\right)}\mathrm{\backslash ]}$$

In which the “logLik” is the log likelihood of a model, and the pseudo R square reflects the magnitude of correlation between variables.

The FDR method was used to get the q-value. We used the threshold q-value < 0.01 and R square > 0 to screen the significant correlation genes.

### Weighted gene co-expression network analysis

To identify gene co-expression modules associated with MASLD, we performed a weighted gene co-expression network analysis (WGCNA) on RNA seq data of MASLD using the R package “WGCNA” (https://CRAN.R-project.org/package=WGCNA). In this study, we chose the soft threshold β = 12 (scale free R^2^ = 0.98). The genes within modules were selected as the candidate hub genes.

### Go and KEGG enrichment analysis

To investigate the biological pathways that might be involved in the occurrence and development of MASLD, MASH and fibrosis, DEGs were subjected to Gene Ontology (GO) and Kyoto Encyclopedia of Genes and Genomes (KEGG) enrichments using the R package “clusterProfiler”. The GO analysis involved three categories, namely molecular functions (MF), cellular components (CC), and biological processes (BP). The threshold was set as q-value < 0.05.

### Protein-protein interaction networks

The protein-protein interaction (PPI) network for DEGs was constructed using STRING, BioGrid, OmniPath and InWeb_IM [12–15]. Only STRING (physical score > 0.132) and physical interactions in BioGrid were used. The MCODE plugin was used to find important modules enriched in the PPI network. Genes in crucial (top score) modules were selected as candidate genes.

### Machine learning-based model training, testing and independent validation

To analyze the feasibility of candidate genes in identifying MASLD, MASH and fibrosis, receiver operating characteristic (ROC) curve prediction was performed according to clinical data (S6 File).

We used bootstrap method for estimating the confidence intervals [16,17]. Briefly, samples were repeatedly extracted 100 times (randomly sampling) from the original sample data. 80% cases were extracted and used for model construction each time. The 95% confidence intervals of AUC, sensitivity and specificity were estimated using 100 times repeated sampling. Hanley Mcneil method was used to test the significant differences between AUC values [18]. Because the negative and positive samples are unbalance in the Fibrosis dataset, over-sampling (also called up-sampling) methods are used to increase the number of negative samples in a minority class. The hyperparameters used for the random forest algorithm were selected using grid search each time, the hyperparameters give the best performance in test dataset are chosen to construct models. The optimized hyperparameters in our study including nestimators and max_features. Other hyperparameters are default in the software. The hyperparameters for the best MASLD diagnosis model are 200 and 20 respectively using grid search.

Several independent MASLD datasets (GepLiver-bulk-05_GSE126848, GepLiver-bulk-09_GSE167523, GepLiver-bulk-13_E-MTAB-6863) are used to verify our MASLD model. There is no information of NAS score or fibrosis stages in these independent datasets, and we cannot find other independent MASH and Fibrosis dataset that containing information of NAS score or fibrosis stages, thus MASH and Fibrosis models are not verified using independent dataset in our study.

## Results

### Correlation of sex and age with MASLD, MASH, fibrosis

The Pearson correlation coefficients of MASLD, MASH, fibrosis with sex were 0.356, 0.236, 0.285 respectively, and the Pearson correlation coefficients of MASLD, MASH, fibrosis with age were 0.247, 0.312, 0.268 respectively (Table 1). The results indicated that age and sex had low correlation with MASLD, MASH and fibrosis, which means these factors will not significantly affect our analysis.

**Table 1:** The Pearson correlation coefficients of MASLD, MASH, fibrosis with sex and age.

Phenotype	Sex	Age
MASLD	0.365	0.247
MASH	0.236	0.312
Fibrosis	0.285	0.268

### Genes, modules, and pathways associated with MASLD

**Fig 1:**
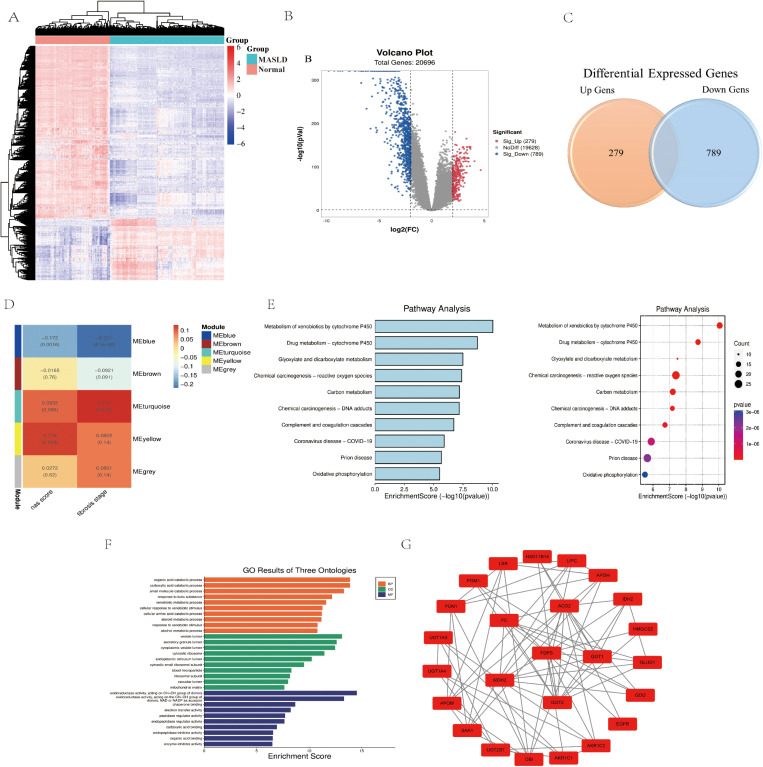
Genes, modules, and pathways associated with MASLD. (A) Heatmap of significantly DEGs between normal and MASLD liver tissues. The color from blue to red represents the progression from low expression to high expression. (B) Volcano plot of differentially expressed genes between Normal and MASLD. The red dots in the plot represents upregulated genes and blue dots represents downregulated genes with statistical significance. Gray dots represent there is no differential expression in those genes. (C) Venn diagram of differentially expressed genes between Normal and MASLD. (D) WGCNA module plot of genes in MASLD. Each row represents a co-expression module. Each column represents a trait attribute. Blue color represents negative correlation and red color represents positive correlation. (E) KEGG pathways of DEGs. (F) Go analysis. The figure represents BP, CC and MF of DEGs. (G) The most significant enriched module in the PPI network.

Total 1,068 DEGs (279 upregulated and 789 downregulated) were screened between MASLD and normal liver tissues. The heatmap of DEGs were presented in Fig 1A, which revealed that MASLD samples can be distinguished from the normal samples. Volcano plots and venn plot shows the distribution of DEGs between MASLD and normal controls (Fig 1B and 1C). These identified DEGs are expected to have possible roles in the development and progression of MASLD.

In WGCNA, a total of 5 co-expression modules were identified in MASLD. The turquoise and yellow modules comprised 1237 positive correlation genes, and the blue modules comprised 421 negatively correlated genes (Fig 1D). Among these genes, a total of 448 genes overlapped with DEGs.

Subsequently, these 448 genes were analyzed by GO and KEGG enrichments to clarify the biological functions. The top 10 enriched GO and KEGG pathways of DEGs in MASLD are listed in Table 2. In GO analysis, most of the top 10 enriched pathways are associated with catabolism and metabolism of organic compounds (Table 2, Fig 1F). In KEGG analysis, most OF TOP 10 enriched pathways are associated with substance (e.g., xenobiotics, drug, carbon) metabolism (Table 2, Fig 1E). The complete result of KEGG, GO (BP, CC, MF) analysis of DEGs for MASLD were summarized in S2 File of Supplemental Digital Content.

**Table 2:** Functional Enrichment Analysis of GO and KEGG Pathways of DEGs in the MASLD, MASH and Fibrosis.

Phenotype	GO			KEGG
	Biological Process	Cell component	Molecular Function	
MASLD	Organic acid catabolic process, carboxylic acid catabolic process, small molecule catabolic process, response to toxic substance, xenobiotic metabolic process, cellular response to xenobiotic stimulus, cellular amino acid catabolic process, steroid metabolic process, response to xenobiotic stimulus, alcohol metabolic process, etc.	vesicle lumen, secretory granule lumen, cytoplasmic vesicle lumen, cytosolic ribosome, endoplasmic reticulum lumen, cytosolic small ribosomal subunit, blood microparticle, ribosomal subunit, vacuolar lumen, mitochondrial matrix, etc	oxidoreductase activity, acting on CH-OH group of donors, oxidoreductase activity, acting on the CH-OH group of donors, NAD or NADP as acceptor, chaperone binding, electron transfer activity, peptidase regulator activity, endopeptidase regulator activity, carboxylic acid binding, endopeptidase inhibitor activity, organic acid binding, enzyme inhibitor activity, etc.	Metabolism of xenobiotics by cytochrome P450, Drug metabolism – cytochrome P450, Glyoxylate and dicarboxylate metabolism, Chemical carcinogenesis – reactive oxygen species, Carbon metabolism, Chemical carcinogenesis DNA adducts, Complement and coagulation cascades, Coronavirus disease - COVID-19, Prion disease, Oxidative phosphorylation, etc.
MASH	positive regulation of leukocyte migration, response to nutrient, chemokine-mediated signaling pathway, response to chemokine, cellular response to chemokine, regulation of leukocyte migration, neutrophil chemotaxis, myeloid leukocyte migration, triglyceride catabolic process, neutrophil migration, etc.	anchored component of external side of plasma membrane, intrinsic component of external side of plasma membrane, anchored component of membrane, anchored component of plasma membrane, lipid droplet, chylomicron, axolemma, external side of plasma membrane, tertiary granule, cell leading edge, etc.	cytokine activity, chemokine activity, receptor ligand activity, signaling receptor activator activity, chemokine receptor binding, long-chain fatty acid transporter activity, apolipoprotein binding, CXCR chemokine receptor binding, glycosaminoglycan binding, alcohol dehydrogenase (NADP+) activity, etc.	Cytokine-cytokine receptor interaction, PPAR signaling pathway, IL-17 signaling pathway, Viral protein interaction with cytokine and cytokine receptor, TNF signaling pathway, Regulation of lipolysis in adipocytes, Glycerolipid metabolism, Chemokine signaling pathway, Drug metabolism – cytochrome P450, Toll-like receptor signaling pathway, etc.
Fibrosis	detection of chemical stimulus involved in sensory perception of taste, detection of chemical stimulus involved in sensory perception of bitter taste, sensory perception of bitter taste, protein K48-linked deubiquitination, covalent chromatin modification, histone modification, regulation of small GTPase mediated signal transduction, peptidyl-lysine modification, positive regulation of GTPase activity, regulation of chromosome organization, etc.	cell leading edge, centriole, site of polarized growth, site of DNA damage, growth cone, PML body, site of double-strand break, chromosomal region, cytoplasmic ribonucleoprotein granule, pericentriolar material, etc.	bitter taste receptor activity, taste receptor activity, ATPase activity, guanyl-nucleotide exchange factor activity, nucleoside-triphosphatase regulator activity, GTPase activator activity, GTPase regulator activity, DNA-dependent ATPase activity, protein serine/threonine kinase activity, small GTPase binding, etc.	Taste transduction, ABC transporters, Signaling pathways regulating pluripotency of stem cells, Phosphatidylinositol signaling system, Lysine degradation, Ubiquitin mediated proteolysis, Thyroid hormone signaling pathway, Hepatocellular carcinoma, Adherens junction, Colorectal cancer, etc.

The PPI network of these 448 genes containing 161 edges was constructed by STRING, which provides critical assessment and integration of protein-protein interactions, including direct (physical) and indirect (functional) correlations (Fig 1G). In the PPI network, the MCODE plugin identified 12 significant enriched modules. 25 genes (*UGT2B7*, *UGT1A9*, *UGT1A4SAA1*, *PON1*, *PGM1*, *PC*, *MDH2*, *LSR*, *LIPC*, *IDH2*, *HSD17B14*, *HMGCS2*, *GOT2*, *GOT1*, *GLUD1*, *GDI2*, *FDPS*, *EGFR*, *DBI*, *APOM*, *APOH*, *AKR1C2*, *AKR1C1*, *ACO2*) in the first score of clusters were selected as the candidate hub genes associated with MASLD. These candidate hub genes were enriched in the pathway of glycolipid metabolism. The AUC values of the 25 candidate genes were showed in Fig 4A, most of which were greater than 0.8.

**Fig 2:**
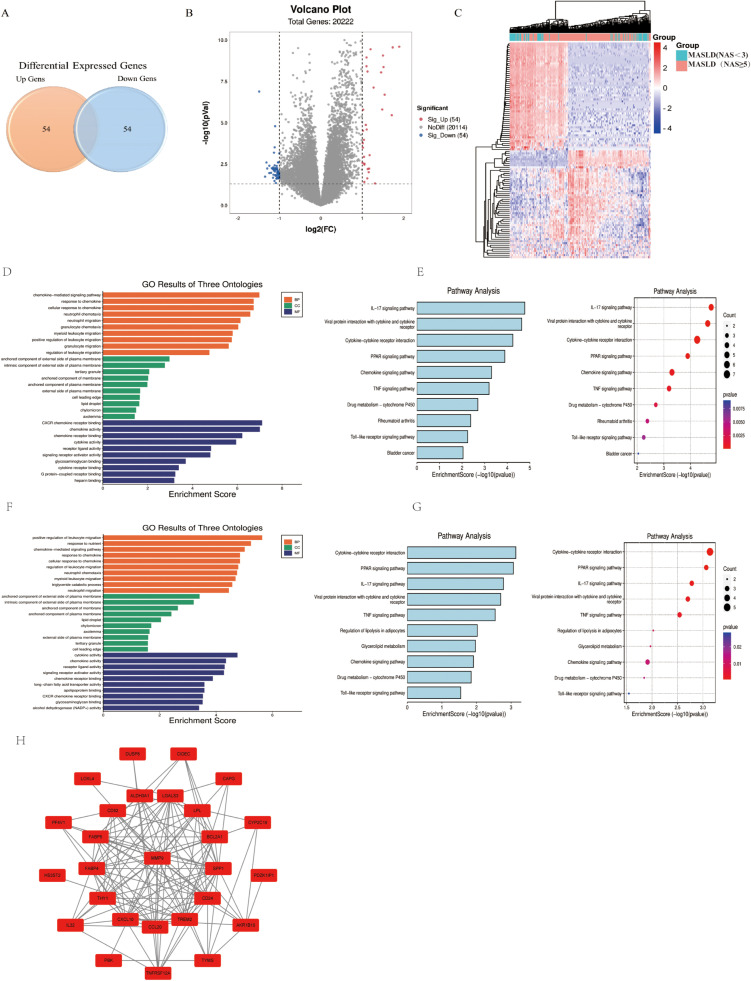
Genes, modules, and pathways associated with MASH. (A) Venn diagram of DEGs with MASH. (B) Volcano plot of DEGs with MASH. The red dots in the plot represents upregulated genes and blue dots represents downregulated genes with statistical significance. Gray dots represent there is no differential expression in those genes. (C) Heatmap of significantly DEGs with MASH. The color from blue to red represents the progression from low expression to high expression. (D) Go analysis. The figure represents BP, CC and MF of the DEGs. (E) The most significant KEGG pathways of the DEGs. (F) Go analysis. The figure represents BP, CC and MF of the DEGs overlapped with those screened by correlation analysis. (G) The most significant KEGG pathways of DEGs overlapped with those screened by correlation analysis. (H) The most significant enriched module in the PPI network.

**Fig 3:**
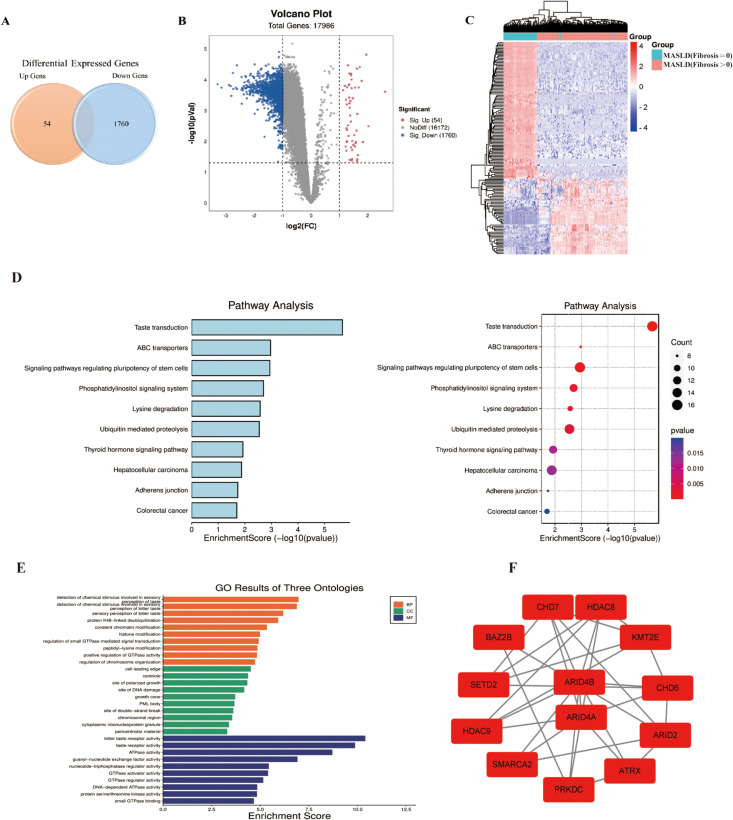
Genes, modules, and pathways associated with the fibrosis in MASLD. (A) Venn diagram of DEGs with the fibrosis. (B) Volcano plot of DEGs with the fibrosis. The red dots in the plot represents upregulated genes and blue dots represents downregulated genes with statistical significance. Gray dots represent no differentially expressed genes. (C) Heatmap of significantly DEGs with the fibrosis. The color from blue to red represents the progression from low expression to high expression. (D) The most significant KEGG pathways of DEGs. (E) Go analysis. The figure represents s BP, CC and MF of the DEGs overlapped with those screened by correlation analysis. (F) The most significant enriched module in the PPI network.

**Fig 4:**
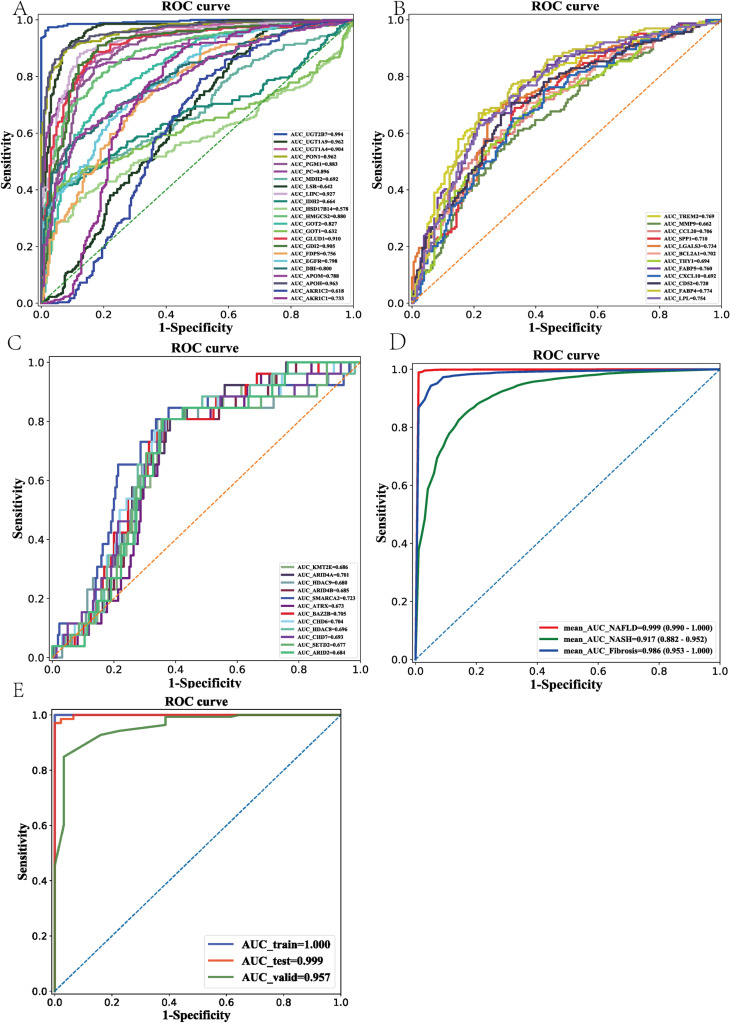
Diagnostic models for MASLD, MASH and fibrosis. (A) ROC curve of each single candidate gene for MASLD diagnosis. (B) ROC curve of each single candidate gene for MASH diagnosis. (C) ROC curve of each single candidate gene for fibrosis diagnosis. (D) ROC curves of models with combined candidate genes for MASLD, MASH and fibrosis diagnoses. (E) ROC curves of models with combined candidate genes for MASLD, MASH and fibrosis diagnoses in independent datasets.

### Genes, modules, and pathways associated with MASH

A total of 108 DEGs (54 upregulated and 54 downregulated) were identified between MASLD samples with NAS ≥ 5 and NAS < 3. The heatmap (Fig 2A) revealed that MASH (NAS ≥ 5) samples can be obviously distinguished from the MAFLD (NAS < 3) samples based on DEGs. Volcano plots and venn plots shows the distribution of DEGs (Fig 2B and 2C). These identified DEGs are expected to have possible roles in the development and progression of MASH.

GO analysis of DEGs in MASH revealed that most of top 10 enriched pathways are linked to chemokine-mediated signaling and immune cells (e.g., leukocyte, neutrophil, granulocyte) migration (Fig 2D). KEGG analysis of DEGs in MASH revealed that most of top 10 enriched pathways are linked to immune-inflammatory regulation (e.g., cytokine, chemokine, IL-17, Toll-like receptor) signaling pathways (Fig 2E). Complete results of the KEGG and GO analyses (BP, CC, MF) for MASH-associated DEGs are provided in S3 File (Supplemental Digital Content).

A total of 2,528 genes were identified significantly correlated with NAS using ordinal logistic regression and 38 genes were overlapped with DEGs. GO analysis of the 38 candidate genes revealed that most of top10 enriched pathways are linked to chemokine-mediated signaling and immune cells (e.g., leukocyte, neutrophil,) migration (Table 2, Fig 2F). KEGG analysis of 38 candidate genes in MASH revealed that most of top 10 enriched pathways are linked to immune-inflammatory regulation (e.g., cytokine, chemokine, TNF, Toll-like receptor) signaling pathways (Table 2, Fig 2G). The complete result of KEGG, GO (BP, CC, MF) analysis of 38 candidate genes for MASH were summarized in S4 File (Supplemental Digital Content).

The PPI network of these 38 genes containing 125 edges was constructed (Fig 2H). The 12 genes with a degree score greater than 10 were identified as candidate hub genes for MASH, including five key genes related to inflammation (*MMP9*, *CXCL10*, *CCL20*, *BCL2A1*, *THY1*), and the remaining eight key genes were related to lipid metabolism (*TREM2*, *LPL*, *CD52*, *SPP1*, *LGALS3*, *CD24*, *FABP4*, *FABP5*). The AUC values of the 12 candidate genes for MASH detection were showed in the range of 0.6–0.8 (Fig 4B).

### Genes, modules, and pathways associated with the fibrosis in MASLD

A total of 1,814 DEGs (54 upregulated and 1,760 downregulated) were identified between MASLD samples with fibrosis stage = 0 and fibrosis stage > 0. The heatmap (Fig 3A) revealed that samples with fibrosis can be obviously distinguished from the samples without fibrosis based on DEGs. Volcano plots and Venn plot shows the distribution of DEGs (Fig 3B and 3C). The identified DEGs are expected to have possible roles in the development and progression of fibrosis.

After the ordinal logistic regression analysis, a total of 8,257 genes that significantly correlated with the fibrosis stage were screened, in which 1,583 genes were overlapped with DEGs. GO analysis of the 1,583 candidate genes revealed that most of top10 pathways are associated with protein deubiquitination, chromatin and histone modification, chemical stimulus etc (Table 2, Fig 3E). KEGG analysis revealed that most of top10 pathways are linked to energy metabolism (ATP/GTP regulation) and cellular responses (signaling, structural remodeling) (Table 2, Fig 3D). The complete result of KEGG, GO (BP, CC, MF) analysis of DEGs for fibrosis of MASLD were summarized in S5 File of Supplemental Digital Content. The enriched pathways were discovered to be predominantly associated with ubiquitin and lipid metabolism.

Protein-protein interaction (PPI) enrichment analysis was performed on these 1,583 genes. Six important modules were enriched in the PPI network. Thirteen genes (*BAZ2B*, *ARID2*, *ARID4B*, *CHD6*, *HDAC8*, *SMARCA2*, *HDAC9*, *SETD2*, *PRKDC*, *ATRX*, *ARID4A*, *CHD7*, *KMT2E*) in the cluster with highest score were selected as the candidate hub genes associated with fibrosis in MASLD (Fig 3F). The AUC values of the 13 candidate genes were showed in Fig 4C.

### Diagnostic models for MASLD, MASH and fibrosis

We constructed three alternative diagnostic models for MASLD, MASH, and fibrosis using 25, 13, and 13 candidate genes, respectively. Using bootstrap methods, the average AUC, sensitivity, specificity for distinguishing MASLD from normal individuals was 0.999 (95%CI: 0.990–1.00), 0.987 (95%CI: 0.951–1.000) and 0.991 (95%CI: 0.960–1.000) respectively. In MASH detection, the average AUC, sensitivity and specificity were 0.917 (95%CI: 0.882–0.952), 0.807 (95%CI: 0.651–0.963) and 0.873 (95%CI: 0.744–1) respectively. In the fibrosis detection, the average AUC, sensitivity and specificity were 0.986 (95%CI: 0.953–1.000), 0.908 (95%CI: 0.897–1.000) and 0.986 (95%CI: 0.925–1.000) respectively (Fig 4D). The AUC difference between MASLD and MASH is significant (*p* = 0.023). No significant difference of AUC is found between MASLD and fibrosis (*p* = 0.32) or between MASH and fibrosis (*p* = 0.071). To assess the generalizability and potential clinical utility of MASLD, the best MASLD model was constructed with grid search method and independent datasets were used to validate the model. The AUC for distinguishing MASLD from normal individuals in train, test and independent validation dataset were 1.000, 0.999 and 0.957 respectively (Fig 4E).

## Discussion

MASLD is a complex disease characterized by the accumulation of fat droplets in liver cells, which manifest as steatosis in the early stages. Ongoing oxidative stress and inflammation can trigger further liver damage that progresses to MASH [7]. Approximate 40 percent of patients with MASH progressively deteriorate and progress to liver fibrosis and cirrhosis [19]. MASH is a key in the progression of MASLD. Besides the abnormal liver lipid deposition, MASH is also accompanied by pathological changes such as hepatocyte swelling and liver inflammation, and the pathogenesis of MASH is still unclear. There are significant challenges in the diagnosis and treatment of MASLD complicated with fibrosis, and the molecular mechanisms underlying the progression of liver fibrosis in patients with MASLD are unclear [20]. The progression of liver fibrosis in patients with MASH remains untreated, and more research is needed to ascertain molecular processes of liver fibrosis in MASH and to identify important therapeutic targets [21,22]. Therefore, it is necessary to further explore the occurrence, pathogenic factors and related mechanisms of MASH, liver fibrosis. Moreover, alternative diagnostic models for MASLD, MASH, and fibrosis are anticipated to be developed through the investigation of genes and pathways associated with their progression, the refinement of pathogenic factors, and the identification of key genes.

Several omics studies have demonstrated that the occurrence of MAFLD is mainly associated with steatosis, while the occurrence of MASH is predominantly related to inflammation and fibrosis. Gene expression profiles in liver tissue change with the progression of MASLD. For example, the expression of lipid metabolism-related genes is significantly increased after hepatic lipodeposition and steatosis, and is also significantly increased after MASH progression or liver fibrosis [23–26]. Expression of genes related to inflammation and fibrosis is also significantly elevated during the progressive phase of MASLD. Therefore, there is a significant correlation between the expression of certain genes in liver tissue and the severity of MASLD disease activity and the stage of clinical progression [23]. Thus, measuring the expression levels of these genes may help determine the clinical subtype or disease severity of MASLD or help to predict the risk of disease progression [27,28].

MASLD related hub genes were found to enrich in pathways related to glycolipid metabolism. MASLD is often correlated with obesity, diabetes mellitus type 2 (T2DM), and other disorders associated with dysfunction in glucose and lipid metabolism. Studies have demonstrated that T2DM predisposes to the development of MASLD through insulin resistance and hyperglycemia, and this may consequently contribute to the risk of cirrhosis and malignant tumors of the liver [29,30]. In fatty liver mice model, downregulation of *Apolipoprotein H* gene was found to aggravate fatty liver and induce gut microbiota dysbiosis by dysregulating bile acids [31]. Liu et al. found a significant inhibitory of *FDPS* gene can attenuate the mouse NASH-related phenotype. Overexpression of *FDPS* in mice causes increased lipid accumulation, inflammation, and fibrosis, while hepatic *FDPS* deficiency protects mice from NASH progression [32].

MASH is considered as an inflammatory subtype of MASLD with steatosis and evidence of hepatocellular damage and interactions among multiple immune cells [33]. Our data found that 38 candidate genes (e.g., *FABP4* and *MMP9*) in MASH were enriched in inflammation and lipid metabolism pathways. A recent study discovered that the expression levels of *FABP4* and *MMP9* can effectively identify patients who are at risk of progressing from MAFLD to MASH, as well as those at risk of advancing from MASH to cirrhosis and HCC, respectively [34]. Zhao [35] et al. further demonstrated that the protein expression level of MMP-9 was significantly increased when exposed to free fatty acids through in vitro experiments on HepG2 cells. In previous studies, it was found that the serum FABP4 level in MASLD was positively correlated with the severity of hepatic steatosis. The expression of *FABP4* was mainly distributed in liver sinusoidal endothelial cells, which was significantly increased in mice with high fat diet [36]. Subsequently, Zhou et al. used flow cytometry to identify macrophages and found that *FABP4* in liver sinusoidal endothelial cells may play a pathogenic role in the course of MASLD by activating NF-κB/p65 signaling to promote CXCL10-mediated macrophage M1 polarization and CXCR3 macrophage infiltration [37].

DEGs in fibrosis were found to enrich in the Lysine degradation, Ubiquitin mediated proteosis pathways, and so on. Lysine acetylation can regulate Smad2 transcriptional activity, which inhibits hepatic stellate cells activation and liver fibrosis [38]. Ubiquitin-mediated proteolysis plays a significant role in the pathogenesis of liver fibrosis, a condition characterized by the excessive accumulation of extracellular matrix proteins. This process involves the tagging of proteins with ubiquitin, marking them for degradation by the proteasome, which is crucial for maintaining cellular homeostasis and regulating various biological processes [39]. In agreement, The E3 ubiquitin ligase RNF41 has been shown to orchestrate macrophage-driven fibrosis resolution and hepatic regeneration [40]. Another study using multi-omics integration emphasized the role of ubiquitination in CCl_4_-induced liver fibrosis [41]. Moreover, the therapeutic role of some ubiquitin-like proteins has been addressed in the context of liver fibrosis, indicating potential targets for therapeutic intervention [42].

Interestingly, we discovered that the development of MASLD, MASH and fibrosis were all associated with the lipid metabolism processes. The discovered pathways (or GO terms) related to the lipid metabolism process were the common ones among MASLD, MASH and fibrosis. Therefore, lipid metabolism may not only involve in the development of MASLD but also may play a synergistic role in MASH and related liver fibrosis. This suggests that the pathways and genes related to lipid metabolism may be one of the key points that we need to pay attention to in the diagnosis, treatment and drug development of MASLD.

Currently, liver biopsy is the gold standard method that can reliably distinguish MASH from MASLD and accurately measure the degree of fibrosis. However, the consistency and accuracy of the diagnosis may be compromised due to the experiential limitations and subjective bias of pathologists [6,43]. For example, the judgment of fibrosis grade and MASH often varies among pathologists [44]. In our study, we established alternative diagnostic models for MASLD, MASH, and fibrosis by integrating RNA expression profiles of a small set of genes, respectively. The gene expression quantification assays, such as gene expression microarray and high-throughput target sequencing, have been standardized, guaranteeing that the outcomes are objective and devoid of human subjective biases. Furthermore, once trained, the model generates precise and consistent results based on specific data inputs. Consequently, the integration of these models with gene expression quantification experiments can contribute to automate, batch, and objectify the diagnostic processes.

It is possible that the samples used in our study are only a partial representation of the MASLD population, which may lead to the loss identification of some other important hub genes. Some batch effects may also exist in our collected datasets and causes misidentification of hub genes. This also means the constructed model that based on the identified hub genes in our study may be not robustness and not have a universal applicability in all MASLD population, especially the MASH and Fibrosis models which lacks the independent validation dataset. Another limitation of our study is we didn’t address the influence of other possible variability (e.g., race, ethnicity) to the MASLD, MASH and fibrosis diseases due to the lack of those information. In our future study, we will collect some MASLD, MASH and Fibrosis samples to verify the robustness and clinical application of our models. We will also pay a close attention to the public datasets, which have detail population demographics and clinic information about NAS score and fibrosis stage, to further validate our models.

In conclusion, our study integrates multiple datasets to identify the hub genes that involved in the development and progression of MASLD, MASH, and fibrosis. Three diagnostic models based on identified hub genes show good performance in the diagnosis of MASLD, MASH, and fibrosis, implicating its potential clinical values in disease diagnosis. However, further validation and refinement of these models is necessary before they can be applied in clinical practice.

## Supporting information

S1 File Supplemental Digital Content. Description of detailed data sources and clinical information.
(XLSX)

S2 File Supplemental Digital Content. Results of GO and KEGG enrichment analysis with DEGs in MASLD.
(XLSX)

S3 File Supplemental Digital Content. Results of GO and KEGG enrichment analysis with DEGs in NASH.
(XLSX)

S4 File Supplemental Digital Content. Results of GO and KEGG enrichment analysis with DEGs that overlapped with screened genes using ordinal logistic regression in NASH.
(XLSX)

S5 File Supplemental Digital Content. Results of GO and KEGG enrichment analysis of DEGs with the fibrosis in MASLD.
(XLSX)

S6 File Description of correlation analysis and ROC analysis. (DOCX)

## Abbreviations

MAFLD: Metabolic dysfunction-associated fatty liver disease
MASLD: Metabolic dysfunction-associated steatosis liver disease
MASH: Metabolic dysfunction-associated Steatohepatitis
NAS: NAFLD Activity Score
GEO: Gene Expression Omnibus
PPI: Protein-protein interaction
GO: Gene Ontology
MF: Molecular Functions
CC: Cellular Components
BP: Biological Processes
KEGG: Kyoto Encyclopedia of Genes and Genomes
ROC: Receiver operating characteristic
