# Supplementary material for: Identification of hub gene for the pathogenic mechanism and diagnosis of MASLD by enhanced bioinformatics analysis and machine learning
Source: PLoS One. 2025 May 28;20(5):e0324972. doi: 10.1371/journal.pone.0324972 (PMC12118866; doi:10.1371/journal.pone.0324972)
Supplement: S6 File — (DOCX) [file pone.0324972.s006.docx]

**Data Analysis**

**Correlation Coefficient Analysis**

Pearson correlation coefficient (r) is calculated by SPSS and it was utilized to calculated and evaluate the correlation between population demographics (e.g. age and gender) and disease outcomes (NAFLD, NASH, and fibrosis). The strength of association was determined by the absolute value of the correlation coefficient (|r|).

Correlation coefficient at 0.8-1 shows a high correlation, correlation coefficient at 0.6-0.4 means a moderate correlation, correlation coefficient at 0.2-0.4 means a low correlation, and correlation coefficient at 0-0.2 means a very low correlation.

**Area under curve**

The Area Under the Curve (AUC) for a Receiver Operating Characteristic (ROC) curve was drew by the matplotlib and sklearn in Python. The detail information about how to draw curve can be found in the official websites <https://scikit-learn.org/stable/api/sklearn.metrics.html> and <https://matplotlib.org/>.

Area under curve (AUC) evaluation criteria: A value between 0.5 and 0.7 presents limited diagnostic value. A value between 0.7 and 0.9 presents a certain amount of diagnostic value. A value greater than 0.9 present a high diagnostic value. In addition, we combined the expression levels of multiple candidate genes to construct three machine learning models.
